# 

**Published:** 1882-04

**Authors:** 


CONTENTS.

Health and Habit..........167
Cold Catching..........168
Ventilation and Fresh Air..........169
Animal Combustion..........170
A Clergyman's Congregation..........172
What is Patent Flour?..........174
The Government Chemist Analyzes two of the Leading Baking Powders..........175
Particles in the Eye..........176
Raising the Hat..........177
Laughing Horses..........177
A very Romantic History..........179
The Story of the Tides..........180
Consumption..........183
Drinking at Meals..........187
To Bring up Children..........189
Health of Employments..........191
Spitting Blood..........193
Small Bed-Chambers..........194
Checking Perspiration..........197
Liquor Drinking..........199
Gratuitous Medical Advice..........200
Temperament Differences..........201
Cold Bathing..........204
Sudden Death..........206
Pure Food..........208
Households Contrasted..........208
Wearing the Beard..........214
Popular Fallacies..........215
Exercise..........218
Health for Children..........220
Well Done..........224

